# Development and Validation of the TELL Score (Pleural Thickening (T), Fluid Echogenicity (E), Loculations (L), and Laterality (L)): A Structured Sonographic Approach to Classifying Pleural Effusions

**DOI:** 10.7759/cureus.96472

**Published:** 2025-11-10

**Authors:** Rishab Rampradeep, Gangadharan Vadivelu

**Affiliations:** 1 Respiratory Medicine, Saveetha Medical College and Hospital, Saveetha Institute of Medical and Technical Sciences (SIMATS) Saveetha University, Chennai, IND

**Keywords:** exudate, pleural effusion, point-of-care ultrasound, sonographic score, tell score, transudate

## Abstract

Introduction: Pleural effusion classification guides management, but Light’s criteria require invasive sampling and laboratory infrastructure and may be prone to misclassifications. A rapid, bedside alternative is needed, particularly in resource-limited settings.

Aim: This study aimed to develop and validate a novel, four-point sonographic scoring system, the TELL (pleural thickening (T), fluid echogenicity (E), loculations (L), and laterality (L)) score, for differentiating pleural effusions at the bedside.

Materials and methods: This is a prospective observational study conducted at Saveetha Medical College Hospital, a tertiary care centre in South India from March 2021 to February 2022. Seventy-four adult patients with pleural effusions underwent thoracic ultrasonography, assessing four “TELL” parameters: pleural thickening (≥3 mm), fluid echogenicity, laterality (unilateral effusions), and presence of loculations. Each positive finding scored one point (range 0-4). Diagnostic performance was compared against Light’s criteria using diagnostic indices and receiver operator characteristic (ROC) curve analysis.

Results: Among 74 participants (mean age 58.7±17.2 years; 59.5% male), 64 effusions (86.5%) were exudates, mainly tuberculosis and malignancy. Sonographic features were more common in exudates: pleural thickening (95.3%), echogenicity (98.4%), loculations (29.7%), and unilateral distribution (85.9%; all p < 0.01). At cut-off ≥2, the TELL score achieved 98.4% sensitivity, 40% specificity, negative predictive value (NPV) 80%, and area under the curve (AUC) 0.79 (95% confidence interval CI: 0.64-0.94)

Conclusions: The TELL score is a rapid, non-invasive screening tool with high sensitivity and NPV, particularly useful when lab confirmation is delayed or unavailable. Multicentre validation and reliability studies in larger cohorts are needed.

## Introduction

Pleural effusion refers to an abnormal accumulation of fluid in the pleural space and remains a common and diagnostically significant finding in clinical practice [1]. Determining the exudative or transudative nature of the effusion is a crucial step in determining its aetiology and guiding management [2]. In India, exudative effusions are predominantly attributable to tuberculosis and malignancy, the former accounting for 44-64% of cases [3-6].

For decades, Light’s biochemical criteria have served as the reference standard to determine the nature of pleural effusions [7]. However, its reliance on paired serum-fluid sampling, invasive thoracentesis, and requirement of lab infrastructure pose challenges in triage settings and resource-constrained environments. Moreover, misclassification is not uncommon in patients with co-existing systemic conditions or diuretic use [8,9].

This makes a case for more accessible and less invasive alternatives that can streamline decision-making and minimize patient discomfort. Thoracic ultrasound (TUS) has emerged as an indispensable tool in respiratory medicine, offering superior sensitivity and specificity compared to chest radiography for pleural effusion detection [10,11]. Several groups have explored the link between the presence of sonographic features such as pleural fluid echogenicity and septations to underlying exudative disease [12].

Yang et al. demonstrated the value of complex sonographic patterns as markers of exudates [13], while subsequent research, including the development of composite scores, has sought to standardise and quantify these observations [14]. However, widespread bedside adoption has been limited, possibly by the complexity or subjectivity of many proposed models and the lack of external validation [15].

The clinical need for a simple, standardised, and validated sonographic tool for pleural effusion classification remains largely unmet. The TELL sonographic score was developed to address this gap; a simple four-point sonographic scoring system, incorporating four easily identifiable features: pleural thickening (T), fluid echogenicity (E), loculations (L), and laterality (L). This study prospectively evaluated the diagnostic accuracy of the TELL score in differentiating exudative from transudative pleural effusions in a real-world Indian cohort and compared its performance to the established biochemical standard.

## Materials and methods

This was a prospective observational study conducted over a 12-month period in the Department of Respiratory Medicine at Saveetha Medical College Hospital, a tertiary care teaching hospital in South India, from March 2021 to February 2022. The study was approved by the Institutional Ethics Committee of the hospital (approval number SMC/IEC/2021/03/013) on March 23, 2021. Written informed consent was obtained from all participants prior to enrolment.

**Figure 1:**
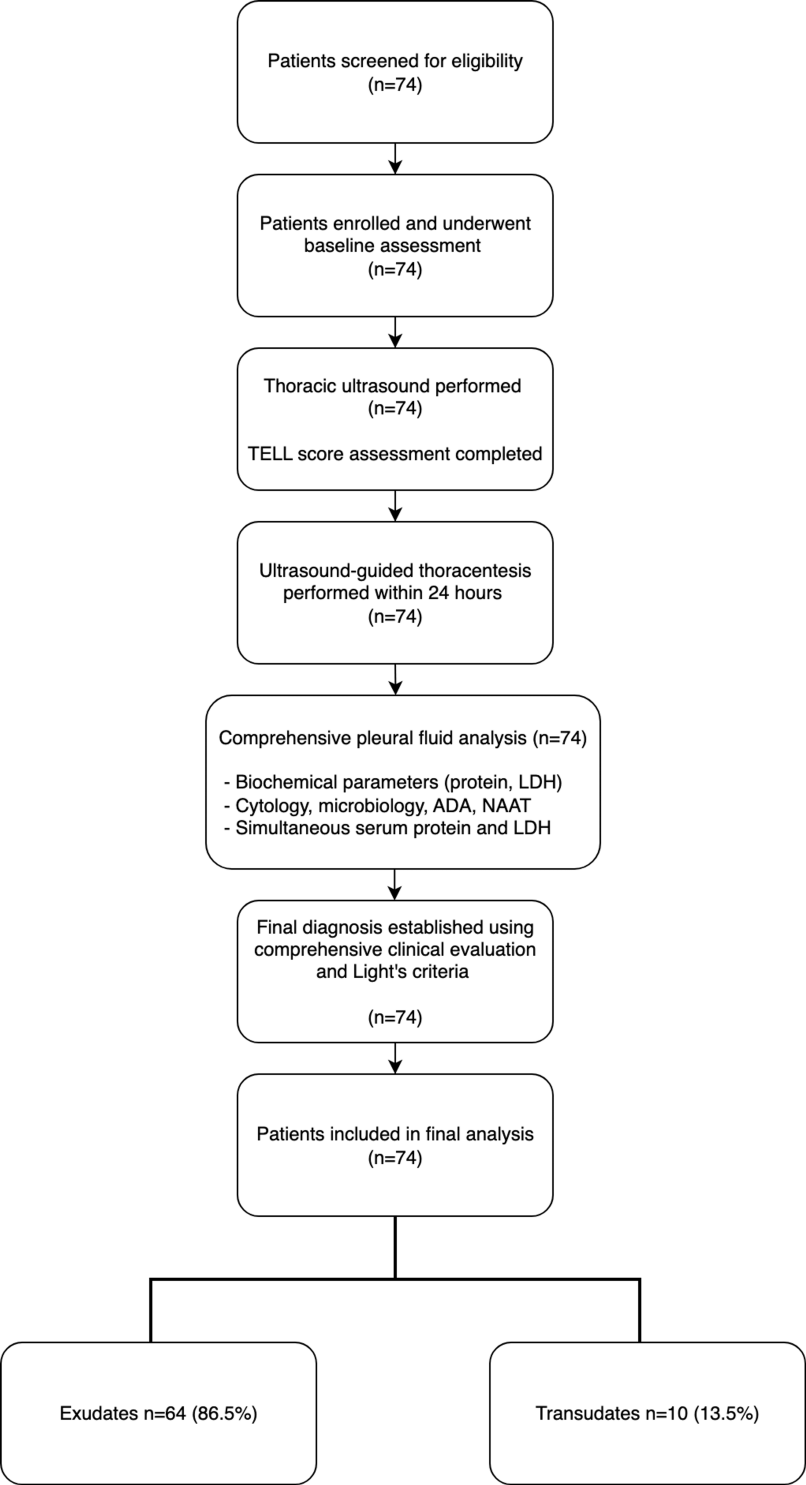
Study flow diagram Patient flow diagram showing enrolment, assessment procedures, and final diagnostic classification in the TELL score validation study. All 74 enrolled patients completed the full diagnostic protocol with no exclusions or dropouts during the study period. Original study flowchart made by the authors

For an expected sensitivity of 90% in detecting exudates, a sample size of 72 patients was determined, allowing for a precision of ±10% and an alpha error of 5% and a total of 74 consecutive patients were ultimately enrolled in the study. All participants were adults admitted to the respiratory medicine department with clinically suspected pleural effusion confirmed by radiological imaging. The participant flow through the study is illustrated in Figure 1.

The inclusion criteria included patients with a definite diagnosis of pleural effusion evidenced by chest radiography or ultrasound imaging, aged 18 years or older, and those who were hemodynamically stable enough to undergo thoracic ultrasound examination prior to thoracentesis or other diagnostic procedures. Exclusion criteria comprised pregnancy, prior thoracentesis for the index effusion, current treatment for pleural effusion before enrolment, traumatic pleural effusion, thoracic wall deformity or diaphragmatic pathology precluding adequate ultrasound visualization, previous chest surgery, bleeding disorders or coagulopathy, and critical illness requiring immediate drainage.

Following detailed history taking and comprehensive clinical examination, all participants underwent chest radiography followed by thoracic ultrasound to determine the presence, laterality, and severity of pleural effusion. This provided initial stratification of effusion severity and guided subsequent management decisions.

Ultrasound examination protocol

All sonographic assessments were performed by the study authors using a standardised protocol, minimizing inter-operator variability and ensuring reproducibility. Ultrasound examinations were performed using a system, equipped with both a 3.5 MHz convex probe and a 10 MHz linear probe. Patients were examined in the sitting position with arms supported to allow optimal access to the posterior thorax. In cases where patients could not sit upright, a lateral decubitus position was used.

Each hemithorax was systematically scanned from the posterior axillary line to the paravertebral region, including anterior, lateral, and posterior zones. The convex probe was used for the initial survey to localize and assess pleural effusions, followed by the linear probe for high-resolution evaluation of the pleura. Pleural fluid volume was estimated using the validated Goecke formula [16], with measurements taken in the erect position using the convex probe placed on the dorsolateral chest wall.

TELL score assessment

The TELL score incorporated four binary parameters, each scoring 0 or 1 point:

Pleural Thickening (T)

Pleural thickness ≥3 mm is measured at end expiration using a linear probe. Measurements were taken at the point of maximal thickness, perpendicular to the pleural surface.

Echogenicity (E)

It is the presence of any internal echoes within pleural fluid, including homogeneous, heterogeneous, or complex patterns. Anechoic (black) fluid scored 0 points.

*Loculation (L)* 

It includes visible septations, fibrin strands, or compartmentalization within the pleural space on B-mode imaging.

Laterality (L)

Unilateral effusion scored 1 point; bilateral effusions scored 0 points.

The total TELL score ranged from 0 to 4 points (Table 1). All ultrasound examinations were performed by the study team.

**Table 1:** TELL score parameters and scoring system Components of the TELL sonographic scoring system for pleural effusion classification. Each parameter is assessed as a binary variable (0 or 1 point) with a total possible score range of 0-4 points.

Parameter	Definition	Scoring
T - Pleural thickening	Parietal pleura thickening ≥3 mm	1 if present; 0 if absent
E - Echogenicity	Any internal echoes within pleural fluid	1 if present; 0 if absent
L - Loculations	Visible loculations/septations/fibrin strands	1 if present; 0 if absent
L - Laterality	Unilateral effusion	1 if unilateral; 0 if bilateral
Total score	Sum of all parameters	Range 0-4 points

The TELL score is a novel sonographic scoring system developed and validated by the study authors as part of this research. No external permission was required.

Thoracentesis and laboratory analysis

Ultrasound-guided thoracentesis was performed within 24 hours using standard sterile technique. Pleural fluid underwent comprehensive analysis including cell count, protein, glucose, lactate dehydrogenase (LDH), adenosine deaminase, microbiological cultures, nucleic acid amplification testing (NAAT) for mycobacteria and fluid cytology. Simultaneous serum protein and LDH levels were obtained for Light’s criteria calculation (Table 2).

**Table 2:** Light’s criteria Biochemical criteria for differentiating exudative from transudative pleural effusions as described by Light et al. [7]. An effusion is classified as exudative if any one criterion is met.

Criterion	Definition
Pleural fluid protein/serum protein ratio	>0.5
Pleural fluid LDH/serum LDH ratio	>0.6
Pleural LDH	>2/3 upper limit of normal serum LDH
Classification	Exudate if ANY criterion is met

Final effusion categorization was established through comprehensive clinical evaluation incorporating patient history, physical examination, imaging studies, pleural fluid biochemistry, microbiology results, and treatment response.

Statistical analysis

Statistical analysis was performed using IBM SPSS Statistics for Windows, Version 26.0 (released 2018, IBM Corp., Armonk, NY). Categorical variables are expressed as frequencies and percentages; continuous variables as mean±SD or median (interquartile range (IQR)) based on normality testing. Diagnostic performance metrics (sensitivity, specificity, positive predictive value (PPV), negative predictive value (NPV), accuracy) were calculated with 95% confidence intervals (CIs). ROC curve analysis determined optimal cut-off points using Youden’s index. Group comparisons used chi-square/Fisher’s exact tests for categorical variables and t-test/Mann-Whitney U for continuous variables. Logistic regression identified independent predictors of exudative effusion. Statistical significance was set at p < 0.05.

## Results

This prospective diagnostic accuracy study enrolled 74 consecutive patients presenting with pleural effusion over a 12-month period from March 2021 to February 2022. The cohort comprised 44 males (59.5%) and 30 females (40.5%), with a mean age of 58.7 ± 17.2 years, demonstrating no significant gender predilection between effusion types (p = 0.087). Final etiological classification revealed that 64 patients (86.5%) had exudative effusions, while 10 patients (13.5%) had transudative effusions, reflecting the predominant burden of infectious and malignant pleural diseases in our setting (Table 3). 

**Table 3:** Baseline demographics and etiological distribution Patient demographics and final diagnostic classification of pleural effusions in the study cohort (n = 74). Etiological categories represent final diagnoses based on comprehensive clinical evaluation including biochemical, microbiological, and cytological analysis.

Parameter	Value (%)
Total patients	74 (100)
Male	44 (59.5)
Female	30 (40.5)
Mean age ± SD (years)	58.7 ± 17.2
Exudative effusions	64 (86.5)
Tuberculosis	37 (50.0)
Malignancy	24 (32.4)
Parapneumonic effusions	3 (4.1)
Transudative effusions	10 (13.5)

Tuberculosis was the leading cause of exudative effusions (37 cases, 50%), followed by malignancy (24 cases, 32.4%), parapneumonic effusions (three cases, 4.1%), and transudative effusions (10 cases, 13.5%). This distribution aligns with the epidemiological pattern of pleural diseases in the Indian subcontinent, where tuberculosis remains a significant health burden.

Age distribution between effusion types showed no significant difference (p = 0.069).

Transudative effusions showed strong associations with systemic conditions (Table 4): hypertension (50% vs. 15.2%, p = 0.01), diabetes mellitus (75% vs. 24.2%, p = 0.003), coronary artery disease (37.5% vs. 3%, p < 0.001), and chronic kidney disease (50% vs. 4.5%, p < 0.001).

**Table 4:** Comorbidity profile by effusion type Comparison of systemic comorbidities between patients with exudative versus transudative pleural effusions. Data presented as number (percentage) with statistical significance determined by Fisher’s exact test. *p < 0.05, **p < 0.01, ***p < 0.001

Comorbidity	Exudates n = 64 (%)	Transudates n = 10 (%)	p-value
Systemic hypertension	10 (15.2)	4 (50.0)	0.01*
Type 2 diabetes mellitus	16 (24.2)	6 (75.0)	0.003**
Coronary artery disease	2 (3.0)	3 (37.5)	<0.001***
Chronic kidney disease	3 (4.5)	4 (50.0)	<0.001***
Congestive heart failure	0 (0)	1 (12.5)	0.004**

Unilateral effusions predominated in exudative cases, occurring in 85.9% of exudates compared to only 40% of transudates (χ² = 15.835, p = 0.001). Among unilateral effusions, right-sided predominance was observed in 45.5% of exudative cases. Conversely, bilateral effusions were significantly more common in transudative cases (75% vs. 15.1%), reflecting the systemic pathophysiology underlying these conditions.

Analysis of individual TELL score components revealed varying discriminatory capabilities (Table 5).

**Table 5:** Diagnostic performance of individual TELL components Individual diagnostic accuracy of each TELL score parameter for identifying exudative pleural effusions. Odds ratios calculated using logistic regression with 95% confidence intervals. *Odds ratio could not be calculated due to zero values in transudate group.

TELL component	Exudates n = 64 (%)	Transudates n = 10 (%)	p-value	Odds ratio (95% CI)
Pleural thickening ≥3 mm	61 (95.3)	3 (30.0)	<0.001	45.7 (8.1-257.8)
Echogenic fluid	63 (98.4)	4 (40.0)	<0.001	94.5 (8.7-1028.3)
Loculations present	19 (29.7)	0 (0)	0.048	Could not be assessed*
Unilateral distribution	55 (85.9)	4 (40.0)	0.006	9.6 (2.4-38.9)

When compared to transudates, exudative effusions demonstrated significantly higher odds ratios (ORs) for all four TELL parameters. Pleural thickening emerged as the most powerful predictor, present in 95.3% of exudative effusions compared to only 30% of transudative effusions (OR 45.7, 95% CI: 8.1-257.8, p < 0.001). This finding supports the pathophysiological understanding that inflammatory processes characteristic of exudative diseases lead to pleural membrane thickening. Fluid echogenicity represented the second most discriminatory feature, with internal echoes observed in 98.4% of exudates versus 40% of transudates (OR 94.5, 95% CI: 8.7-1028.3, p < 0.001). This high sensitivity reflects the increased protein content and cellular debris characteristic of exudative effusions.

Loculations showed 100% specificity when present, being identified in 29.7% of exudative effusions while remaining absent in all transudative cases (p = 0.048). However, the relatively low prevalence of loculations limited its overall discriminatory power. The laterality component, defined as unilateral distribution, strongly favoured exudative etiology (OR 9.6, 95% CI: 2.4-38.9, p = 0.006), reinforcing the clinical principle that bilateral effusions more commonly suggest systemic causes.

The distribution of TELL scores exhibited a clear bimodal pattern distinguishing exudative from transudative effusions (Table 6).

**Table 6:** Distribution of TELL scores by effusion type Frequency distribution of TELL scores stratified by the final effusion classification. Data demonstrate the discriminatory capability of the scoring system with median values and interquartile ranges. Statistical comparison by Mann-Whitney U test (U = 624; p < 0.001).

TELL score	Exudates n = 64 (%)	Transudates n = 10 (%)	p-value
Score 0	0 (0)	2 (20.0)	-
Score 1	1 (1.6)	6 (60.0)	-
Score 2	11 (17.2)	2 (20.0)	-
Score 3	32 (50.0)	0 (0)	-
Score 4	20 (31.3)	0 (0)	-
Median (IQR)	3.0 (3.0-4.0)	1.0 (1.0-2.0)	<0.001

No exudative effusion achieved a score of 0, while 20% of transudates scored 0. At score 1, only 1.6% of exudates were found compared to 60% of transudates. Score 2 represented the overlap zone, with 17.2% of exudates and 20% of transudates falling into this category. Conversely, 81.5% of exudative effusions scored 3 or 4, compared to no transudates achieving these higher scores. The median TELL score was significantly higher in exudates: 3.0 (IQR: 3.0-4.0) versus 1.0 (IQR: 1.0-2.0) in transudates (p < 0.001).

When stratified by specific etiologies, malignant effusions demonstrated the highest mean scores at 3.08, followed by parapneumonic effusions (2.66) and tuberculous effusions (2.54). Among transudative effusions, the mean score was 1.2, validating the utility of the scoring system for distinguishing between effusion types.

**Figure 2:**
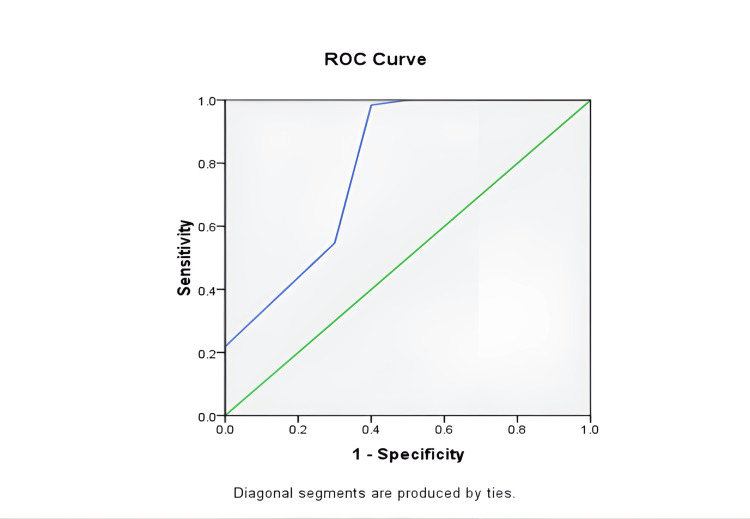
ROC curve analysis Receiver operating characteristic (ROC) curve demonstrating the diagnostic performance of the TELL score. Area under the curve (AUC) = 0.79 (95% CI: 0.64-0.94), indicating good discriminatory ability. Optimal cut-off determined by Youden’s index.

Receiver operating characteristic curve analysis yielded an area under the curve of 0.79 (95% CI: 0.64-0.94) for the TELL scoring system, indicating good discriminatory ability (Figure 2).

Analysis using Youden’s index identified TELL score ≥2 as the optimal cut-off point (Table 7).

**Table 7:** Diagnostic performance at different TELL score cut-offs Sensitivity, specificity, positive predictive value (PPV) and negative predictive value (NPV) of the TELL scoring system at various cut-off thresholds for detecting exudative pleural effusions. Confidence intervals (CI) calculated using the Wilson score method.

Cut-off	Sensitivity (95% CI)	Specificity (95% CI)	PPV (95% CI)	NPV (95% CI)
≥1	100.0% (94.4-100.0)	20.0% (2.5-55.6)	88.9% (76.5-93.7)	100.0% (15.8-100.0)
≥2	98.4% (91.6-99.7)	40.0% (12.2-73.8)	91.3% (76.7-93.2)	80.0% (28.4-99.5)
≥3	81.3% (69.5-90.0)	100.0% (69.2-100.0)	100.0% (93.2-100.0)	45.5% (24.4-67.8)

At this threshold of score ≥2, the system achieved a sensitivity of 98.4% (95% CI: 91.6-99.7%), with a specificity of 40% (95% CI: 12.2-73.8%) for detecting exudative effusions. The PPV reached 91.3% (95% CI: 76.7-93.2%), while the NPV was 80% (95% CI: 28.4-99.5%), resulting in an overall diagnostic accuracy of 90.5%.

Alternative cut-off values demonstrated the trade-off between sensitivity and specificity. At a TELL score ≥1, perfect sensitivity (100%) was achieved, but with very low specificity (20%). At a TELL score cut-off ≥3, specificity improved dramatically to 100% while sensitivity decreased to 81.3%, with a PPV of 100% and NPV of 45.5%. This flexibility allows clinicians to adjust the cut-off based on their clinical priorities, using ≥2 for screening with high sensitivity or ≥3 when specificity is paramount.

The performance characteristics of the TELL score reflect its design as a highly sensitive screening tool. This makes the TELL score particularly valuable as a rule-out test for exudative pathology, making it particularly suitable for rapid triage in resource-limited settings where immediate biochemical analysis is often unavailable. The high sensitivity ensures that virtually no exudative effusion requiring urgent intervention would be missed, while the moderate specificity reflects the expected overlap in sonographic features between some chronic transudative and exudative processes.

## Discussion

Our findings confirm that a compact, four-parameter ultrasound scoring system can reliably screen for exudative pleural effusion in an Indian population, where tuberculosis and malignancy dominate the etiological spectrum. At the optimal threshold ≥2, the TELL score demonstrated exceptional sensitivity of 98.4% and NPV of 80%. This performance profile establishes TELL as a highly effective rule-out test, particularly valuable in resource-limited settings where immediate laboratory support may be unavailable and thoracentesis carries procedural risks. Our sensitivity (98.4%) significantly surpasses Yang et al.’s 93% [13]. Compared to the relatively complex system developed by Mutlu and colleagues (84% sensitivity, 75% specificity) [14], TELL uses four binary parameters, maintaining sensitivity while improving bedside practicality by condensing the assessment to four binary observations. Pleural thickening ≥3 mm independently predicted exudative effusions with an odds ratio (OR) of 45.7 (95% CI: 8.1-257.8), consistent with computed tomography and thoracoscopic studies demonstrating that parietal pleural thickening almost invariably indicates inflammatory processes characteristic of exudates [10,11].

Fluid echogenicity was identified in 98.4% of exudates compared to only 40% of transudates. This observation mirrors the high echogenicity rates described by Asciak et al. [12] and validates its diagnostic utility despite earlier reports questioning its specificity [15]. The high sensitivity of echogenicity reflects the fundamental biochemical differences between exudates and transudates, where increased protein concentration and cellular components in exudates create acoustic interfaces that generate internal echoes on ultrasound examination.

Loculations were observed in 29.7% of exudative effusions, while remaining absent in all transudative cases. This finding aligns with large cohort studies demonstrating that any septated or complex effusion carries a ≥90% probability of being exudative [2]. The formation of loculations represents an advanced inflammatory response with fibrin deposition and adhesion formation, processes exclusive to exudative pathophysiology.

The moderate specificity of 40% observed in our study was primarily attributable to transudative effusions, particularly those related to diuretic-treated heart failure that developed some internal echoes. This phenomenon represents a well-recognized pitfall that similarly affects the specificity of Light's criteria [7,8]. However, given that TELL is designed as a screening tool, we believe that its value should be judged primarily based on the sensitivity and NPV. In this regard, TELL compares favourably with other ultrasound-based algorithms: studies have shown that no single sonographic feature achieved >60% sensitivity for exudate detection [9], while specialized image-processing approaches required software unavailable at most point-of-care ultrasound stations [15].

Clinical integration and practical application

We propose a three-phase implementation strategy for the TELL score: Phase 1 (Screening): TELL score 0-1 → Monitor and optimize treatment for systemic causes of effusion; Phase 2 (Intermediate): TELL score 2 → Clinical judgment based on pre-test probability; Phase 3 (High suspicion): TELL score 3-4 → Proceed with thoracentesis.

This approach offers particular advantages in emergency departments and resource-constrained healthcare settings where point-of-care ultrasound is increasingly available but laboratory turnaround times remain prolonged [11]. The ability to rapidly identify patients unlikely to have exudative effusions could prevent unnecessary invasive procedures, reduce patient discomfort, and optimize healthcare resource allocation.

Study limitations and considerations

The small number of transudative effusions (n = 10) limits the precision of specificity estimates and generalizability of our findings. The predominance of exudative effusions (86.5%) reflects the referral patterns to our respiratory medicine department, where infectious and malignant pleural diseases are primarily managed. This may limit generalizability to settings with different patient populations or referral patterns. Future multi-centre studies incorporating patients from cardiology and nephrology departments, for instance, would provide broader representation of transudate etiologies. A limitation of this study is the absence of original ultrasound images demonstrating the TELL score parameters. During the study period, our ultrasound equipment did not support image export functionality, limiting the availability of study cohort images. Future studies should incorporate direct image documentation to enhance clinical validation and visual clarity.

The single-centre design may limit external validity and may not reflect disease patterns in other geographical regions [1,4]. Inter-observer reliability was not formally assessed; although the four TELL components are designed to be objective, reproducibility studies among operators with varying ultrasound experience should be conducted in future investigations.

Future multi-centre studies with larger, more diverse populations will be essential for confirming these findings.

## Conclusions

The TELL score represents a simple, reproducible, and sensitively optimized alternative to biochemical testing alone for initial pleural effusion triage. Its implementation could reduce unnecessary thoracenteses, accelerate clinical decision-making, and redirect laboratory resources toward cases requiring definitive biochemical characterization. These advantages are particularly relevant where point-of-care ultrasound has become standard practice and rapid diagnostic algorithms are increasingly valued for their ability to improve patient flow and resource utilisation.
